# Efficacy and safety of office-based diode laser ablation for recurrent low-grade non-muscle-invasive bladder cancer under local anaesthesia: A pilot study

**DOI:** 10.1080/20905998.2024.2381816

**Published:** 2024-07-18

**Authors:** Ibrahim A. Khalil, Nagy Younes, Alaeddin Badawi, Khalid Al Rumaihi

**Affiliations:** Department of Urology, Urology Oncology Section, Hamad Medical Corporation, Doha, Qatar

**Keywords:** Bladder cancer, non-muscle invasive, recurrent, laser ablation, TULA

## Abstract

**Introduction:**

Low-grade tumors account for approximately 50% of non-muscle invasive bladder cancer (NMIBC) with recurrence rates between 46% and 62%. Management of NMIBC recurrence typically involves transurethral resection of bladder tumor (TURBT) under general or regional anesthesia, which carries perioperative risks and considerable healthcare costs due to repeated procedures. Therefore, less invasive treatments such as office-based laser ablation, which aim to manage recurrences and reduce inpatient procedures without compromising oncological control, are needed.

**Objectives:**

This study aims to assess the efficacy and safety of office-based diode laser ablation for treating recurrent NMIBC under local anesthesia and to evaluate the influence of tumor size on treatment outcomes.

**Methods:**

A retrospective analysis was conducted on patients with recurrent low-grade NMIBC who underwent office-based diode transurethral laser ablation (TULA) under local anesthesia between 2021 and 2022.

**Results:**

A total of 30 patients were included, with a mean age of 55 (±12) years. The mean original tumor size was 2.82 (±2.59) cm The mean recurrent tumor size was 1.15 (±0.88) cm, with a median of two recurrent tumors (range 1–20). The recurrence rate post-ablation for the entire cohort was 70%, with a median post-ablation recurrence duration of 5 months. The recurrence rate post-TULA was significantly higher in patients with an ablated tumor size of more than 1 cm compared to those with a tumor size of less than 1 cm (86.6% vs. 53.3%, *p* = 0.046). None of the patients experienced tumor progression, with a median follow-up duration of 12 months. Patients tolerated the procedure well, reporting only mild pain, and there were no complications greater than grade 1 on the Clavien-Dindo classification.

**Conclusion:**

Office-based diode laser ablation is a safe, effective, and well-tolerated alternative for treating recurrent low-grade NMIBC with a low volume, less than 1 cm, under local anesthesia.

## Introduction

Non-muscle-invasive bladder cancer (NMIBC) stands as the most prevalent subtype among bladder neoplasms, constituting approximately 70–75% of diagnosed cases [1]. This classification includes a range of urothelial carcinoma (UC) grades, which encompass low-grade and high-grade papillary UC, as well as urothelial carcinoma in situ. The tumor grading system serves as a pivotal prognostic factor, playing a decisive role in predicting the likelihood of recurrence and progression in bladder cancer [2].

Notably, low-grade tumors make up around 50% of NMIBC cases. Despite a relatively low incidence of progression to muscle-invasive bladder cancer (MIBC) and bladder cancer-specific mortality, the long-term recurrence rates are notably higher, ranging from 46% to 62%, particularly in cases involving large or multifocal tumors [3]. This underscores the significance of managing low-grade tumors, with a primary focus on minimizing recurrence rates, extending time to recurrence, alleviating patient discomfort, and reducing healthcare expenditures, all without compromising oncological control [4].

Transitioning from the broader context to specific treatment modalities, conventional transurethral resection of the bladder tumor (TURBT) emerges as the most widely used modality for treating bladder cancer recurrence globally [1]. However, TURBT is not without complications, including bleeding and bladder perforation [5]. Furthermore, the necessity for either spinal or general anesthesia introduces its own set of morbidities and mortalities, particularly in elderly patients [6,7]. Nevertheless, the repeated use of TURBT contributes to the high cost associated with treating bladder cancer, making it one of the most expensive cancers from diagnosis to death [8].

These challenges underscore the pressing need for alternative modalities that not only decrease morbidity and mortality but also address the economic burden without compromising oncological outcomes. Laser therapy has emerged as a promising alternative for the management of recurrent bladder tumors, demonstrating comparable oncological outcomes and a superior safety profile [9–12]. In light of this, the aim of the current study is to evaluate the efficacy and safety of office-based diode laser ablation for the treatment of recurrent low-grade NMIBC under local anesthesia. Additionally, the study aims to compare the efficacy of this approach in both small and large recurrent tumors, providing valuable insights into its potential as a comprehensive treatment strategy.

## Methodology

This is a retrospective cohort study involving patients diagnosed with recurrent NMIBC treated with Transurethral Laser Ablation (TULA) using a diode laser in the outpatient flexible cystoscopy unit at a single center between January 2021 and December 2022. Patients screened were those who underwent TULA; we included patients with primary Ta low-grade NMIBC who had two recurrences or more before the ablation and excluded patients with invasive bladder cancer, carcinoma in situ, and high-grade tumors. Data collected from electronic records included age, gender, smoking history, and comorbidities, as well as details about the original tumor, such as size, number and histology. Additionally, information on tumor recurrence features, such as recurrent tumor size, number of recurrences, date of recurrences, histology of recurrences, any tumor progression, and pain during the TULA procedure according to the numerical pain score [13], was collected. Patients were then divided into two groups based on the mean size of tumor recurrence that was ablated according to the risk stratification with a cutoff of 1 cm [14]. This study has been reviewed and approved by the Institutional Review Board (IRB) under approval number MRC-01-24-199, ensuring adherence to ethical guidelines and participant safety protocols.

## Procedure description

**Figure 1.**
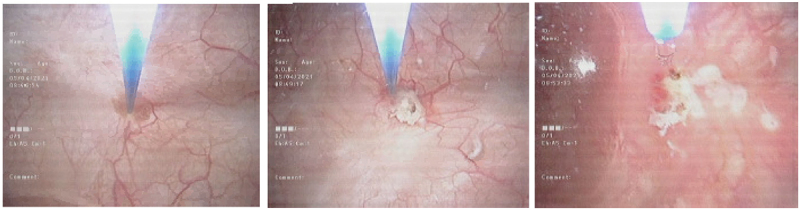
TULA using a diode laser with a 200-micron fiber performed via flexible cystoscopy.

In the outpatient flexible cystoscopy unit, the patient was positioned in the supine position. Prior to cystoscopy, a 20 ml intraurethral instillation of 2% lidocaine gel was administered [15]. A flexible cystoscope with Narrow Band Imaging (NBI) was used to localize tumors and field changes. Subsequently, a biopsy of the lesion was conducted to monitor for any progression. Following the biopsy, laser ablation was performed on all tumors and suspicious areas using the biolitec® LEONARDO® diode laser machine through a 200-micron fiber (Figure 1). Saline with 0.01% lidocaine (100 mg in 1 L NS) was used for irrigation during the ablation [16]. Post-ablation, the patient was monitored for 2 h for haematuria, retention of urine, or other complications and then discharged home.

## Data analysis

Numerical data were presented as mean (standard deviation) for normally distributed variables and median (interquartile range) for skewed variables. Qualitative data were summarized using frequencies and percentages. For quantitative variables, the Unpaired T test was used to compare means, while the Mann-Whitney U test was used to compare medians between the two groups. For qualitative variables, the chi-square test was employed to compare proportions. We used Kaplan–Meier analysis and the log-rank test for comparing time-dependent events. A *p* value of 0.05 was used to identify the significance level of all statistical tests.

## Results

A total of 63 patients were screened, with 30 included, all presenting with primary bladder Ta low-grade urothelial carcinoma with recurrent tumors. The mean age was 55 (±12.03) years, and the male-to-female ratio was 3.2:1. The mean original tumor size was 2.82 (±2.59) cm. The mean recurrent tumor size was1.15 (±0.88) cm, with a median of two recurrent tumors (range 1–20). The recurrence rate post TULA for the entire cohort was 70%, with a median recurrence duration of 5 months, none of the patients experienced tumor progression with a median follow-up duration of 12 months. Patients tolerated the procedure with mild pain with a mean of 1.56 (±0.67) on numerical pain score and there were no complications of more than grade 1 Clavien-Dindo classification in the whole cohort mainly (Table 1).

**Table 1.** Basic patient demographics, and characteristics of original tumor and ablated recurrence.

	Total	Ablated tumor size <1 cm	Ablated tumor size >1 cm	*p* value^a^
Number of patients	30	15	15	
Mean Age, years (SD)	55 (±12.03)	56.94 (±11.39)	53.05(±12.74)	0.19
Gender				0.66
Male	23	11	12	
female	7	4	3	
Comorbidities				0.14
None, n (%)	17(56.6%)	8 (53.3%)	9(60%)	
Diabetes mellites, n (%)	0	0	0	
Hypertension, n (%)	3 (10%)	2(13.3)%	1(6.7%)	
Diabetes mellites and hypertension, n (%)	10 (33.3%)	5 (16.7%)	5 (16.7%)	
Smoking, n, (%)	15 (50%)	7 (46%)	8(53%)	0.71
Original tumor size cm, mean (SD)	2.82 (±2.59)	2.61(±2.39)	3.04 (±2.83)	0.38
Original tumor number, median (IQR)	1(3)	1(1.5)	1 (4)	0.34212
Ablated tumor size cm, mean (SD)	1.15 (±0.88)	0.53 (±0.28)	1.77 (±0.86)	<0.0001
Ablated tumor number, median (IQR)	2 (2)	2 (1)^a^	2 (2.5)	0.097
Recurrence rate^b^, n,(%)	21 (70%)	8(53.3%)^b^	13(86.6%)	0.046

^a^*p* values comparing patients based on the size of tumor recurrence.

^b^Recurrence rate post TULA procedure.

To compare patients based on the size of recurrent tumor ablated tumor, a cut-off of 1 cm was used. A total of 15 patients were in each group. We found that the recurrence rate post TULA was significantly higher in patients with ablated tumor of more than 1 cm with about 86.6% recurrence rate, compared to 53.3% in patients with ablated tumor size less than 1 cm (*p* = *0.046*). Apart from tumor size we did not found and significant difference between the possible risk factors for tumor recurrence post ablation, including patient demographics, smoking status, original tumor characteristics and even the number of recurrent tumors for which ablation was done, the only significant difference was in the size of the recurrent tumor that was ablated *(p < 0.0001)* (Tables 1 and 2).

**Table 2.** TULA procedure outcomes.

	Total	Ablated tumor size <1 cm	Ablated tumor size >1 cm	*p* value^a^
Number of patients	30	15	15	
Recurrence rate^b^, n,(%)	21 (70%)	8(53.3%)	13(86.6%)	0.046
Pain score^c^, (SD)	1.56 (±0.67)	1.53(±0.74)	1.6(±0.63)	0.39
Median recurrence duration^d^, months (range)	5 (2–18)	4 (3–18)	6 (2–18)	0.46
Median follow up duration, months^e^ (range)	12 (4–21)	10 (4–21)	15 (7–21)	0.24

^a^*p* values comparing patients based on the size of tumor recurrence.

^b^Recurrence rate post TULA procedure.

^c^Pain score During TULA measured by numerical pain score.

^d^Median duration for recurrence post TULA procedure.

^e^Median follow up duration post TULA procedure.

The outcomes of the TULA procedure are listed in Table 2, patients tolerated the procedure with mild pain in both groups. While the overall recurrence rates differ between the groups, the median time to recurrence post ablation does not show a statistically significant difference *(p = 0.46).*

## Discussion

The current standard treatment for recurrent NMIBC is TURBT [17]. However, due to the frequent recurrence (up to 62% of cases) observed in low-grade NMIBC, multiple and frequent TURBTs are often necessary to control tumor recurrences, resulting in higher costs and risks for patients [3,5–8]. The use of holmium and thulium lasers in the management of NMIBC has been found to be safe and efficient [18–20]. Additionally, the use of a diode laser for the en bloc resection of NMIBC has been found to be safe and efficient [21]. Although the European Urology Association guideline lists laser fulguration or vaporization as an option for the treatment of small papillary recurrences in patients with Ta low-grade NMIBC [1], the utilization of a diode laser in the management of recurrent NMIBC with multiple recurrences in outpatient settings under local anesthesia is not well established in the literature.

The recurrence rate of NMIBC post-resection or ablation varies according to multiple factors such as multiplicity and the number of tumors, time to recurrence, and whether the patient’s tumor is primary or recurrent [22,23]. It has also been found that with each recurrence, the chances for another recurrence increase. For instance, the risk of recurrence increases by 4.5 times after the second recurrence [24]. This finding is consistent with our cohort, which showed a recurrence rate of 70% post-TULA, as all our patients had at least two recurrences before ablation, which could contribute to the higher recurrence rate in theentire cohort. This is also evident for short recurrence duration in the subsequent recurrences, as it was found that the median time becomes shorter with each recurrence. Bryan et al. found that a second recurrence is associated with a recurrence duration of 6.6 months [25]. As seen in our study, a median recurrence duration of 5 months post-ablation, whichcould also be attributed to the fact that all of our patients had two previous recurrences or more.

One of the major factors for tumor recurrence is the tumor size, as smaller tumors have a lower recurrence rate [14,22,23]. We found that patients with smaller recurrences less than 1 cm behaved better in terms of recurrence rate after TULA, but it did not affect the recurrence duration. Regarding progression in our cohort, larger tumor size was not associated with a higher risk for progression, as there was no progression to a higher stage or grade throughout the whole cohort. This finding is consistent with Jancke et al.‘s study, as they found that tumor size affects recurrence rate but not progression [26]. Other risk factors for recurrence, such as gender, multiplicity of original tumors, and smoking status [22,23], were not found to affect the recurrence rate in this study. This could be attributed to the small sample size.

The safety of the laser is well established with a lower complication rate when compared to electrocautery resection in bladder masses [27,28]. In our study, there were no major complications apart from mild pain associated with the procedure and self-limited hematuria. There were no admissions or second interventions required for any complications. Nevertheless, the whole procedure is done in the outpatient settings under local anesthesia, which reduces the cost of managing recurrent NMIBC [29]. Adding to this, most patients with bladder cancer are elderly with multiple comorbidities, which increases the risks of general and even spinal anaesthesia in such age group [6,7]. For those reasons, there an increased number of transurethral procedures are done now in outpatient setting under local anaesthesia and such prostate water vaporization, laser lithotripsy of bladder stones and laser ablation of bladder tumors [30,31], which marks the change away from the general and spinal anaesthesia whenever possible.

This study provides evidence of the feasibility, safety, and efficacy of diode laser ablation of recurrent NMIBC under local anesthesia, with comparable outcomes to electrocautery resections, which carry a higher risk and cost for patients. Our study has some limitations as it is a retrospective study with a small sample size. Further research with larger sample sizes and longer follow-up durations is needed to support the evidence of the efficacy and safety of outpatient diode laser ablation for recurrent NMIBC.

## Conclusion

Office-based diode laser ablation presents a safe, effective, and well-tolerated alternative for treating recurrent low-grade NMIBC with a low volume, less than 1 cm, under local anesthesia. Future research is needed to provide more insight into the prognostic factors and outcomes.

## Data Availability

Data available on request from the corresponding author.
